# Weekly Training Load across a Standard Microcycle in a Sub-Elite Youth Football Academy: A Comparison between Starters and Non-Starters

**DOI:** 10.3390/ijerph191811611

**Published:** 2022-09-15

**Authors:** José E. Teixeira, Luís Branquinho, Ricardo Ferraz, Miguel Leal, António J. Silva, Tiago M. Barbosa, António M. Monteiro, Pedro Forte

**Affiliations:** 1 Research Centre in Sports Sciences, Health and Human Development, 5001-801 Vila Real, Portugal; 2 Departamento de Desporto e Educação Física, Instituto Politécnico de Bragança, 5300-253 Bragança, Portugal; 3 Department of Sports, Higher Institute of Educational Sciences of the Douro, 4560-708 Penafiel, Portugal; 4 Department of Sports Sciences, University of Beira Interior, 6201-001 Covilhã, Portugal; 5 Department of Sports, Exercise and Health Sciences, University of Trás-os-Montes e Alto Douro, 5001-801 Vila Real, Portugal

**Keywords:** workload, recovery, starting status, periodization, youth

## Abstract

Compensatory training sessions have been highlighted as useful strategies to solve the differential weekly training load between the players’ starting status. However, the influence of the players’ starting status is still understudied in sub-elite youth football. Thus, the aim of this study was to compare the weekly training load on a standard microcycle in starters and non-starters of a sub-elite youth football academy. The weekly training load of 60 young sub-elite football players was monitored during a 6-week period using an 18 Hz global positioning system (GPS), 1 Hz telemetry heart rate, rating of perceived exertion (RPE), and total quality recovery (TQR). The total distance (TD) covered presented a significant difference between starters and non-starters with a moderate effect (*t* = −2.38, Δ = −428.03 m, *p* = 0.018, *d* = 0.26). Training volume was higher in non-starters than in starter players (TD_Starters_ = 5105.53 ± 1684.22 vs. TD_Non-starters_ = 5533.56 ± 1549.26 m). Significant interactive effects were found between a player’s starting status, playing time, and session duration in overall training load variables for within (F = 140.46; η^2^ = 0.85; *p* < 0.001) and between-subjects (F = 11.63 to 160.70; η^2^ = 0.05 to 0.76; *p* < 0.001). The player’s starting status seems to only influence the training volume in sub-elite youth football, unless one considers the covariance of the playing time and session duration. Consequently, coaches should prioritize complementary training to equalize training volume and emphasize similar practice opportunities for non-starters. Future studies should evaluate the gap between training and match load, measuring the impact of recovery and compensatory sessions.

## 1. Introduction

Training load monitoring has been widely reported in youth football research [1,2]. Continuous training monitoring allows the measurement of the players’ physical and physiological demands, allowing them to express their changes in performance and well-being [3,4]. Currently, analyzing and monitoring the weekly training load has become faster and easier to use due to advancements in tracking system applications [5,6]. Thus, the training representation and the game model can be quickly individually tailored through training load monitoring strategies [7,8]. Although most of the evidence has been produced in elite youth football, recently some studies have applied training load strategies in sub-elite cohorts [9,10,11]. Load variation over a standard microcycle in sub-elite football players seems to be influenced by week type, player’s starting status, playing position, training mode, maturation status, and match-related contextual variables [1,11]. Previous studies have reported a high intra-week variation with a low inter-week variation across a standard microcycle in a sub-elite youth football team [10]. When comparing elite and sub-elite football contexts, several differences have been reported in training intensity and patterns [10,11]. However, the player’s starting status is still poorly studied in sub-elite youth training, with few reports in elite contexts [12,13,14,15].

For these reasons, the need inevitably arises to make an adjustment to the training loads of starters and non-starters, as after a game some players may need a complete rest period [16] or regeneration [17], while others must follow their normal training schedule [16,17] or have complete compensatory sessions [18]. In this regard, a recent study indicates that it may be beneficial to use small-sided games (SSG) to control the imposed training load. In fact, even though players can perform the same type of SSG format, there seems to be evidence that the choice of training method (i.e., fractional or continuous) and recovery time between repetitions with the use of the fractional method results in increases and decreases in imposed training loads, respectively [17,18,19]. Based on the results of these authors, starters should perform continuous SSG formats to decrease training load responses, while non-starters should perform fractional formats with short recovery periods to increase training load responses, thus compensating for the difference in game load between players (compensatory training) during the weekly training microcycle [17]. In this way, SSG can be seen as a powerful tool to ensure that starter and non-starter players achieve the goals set by the coach for the training session (e.g., distances covered, different speed zones, accelerations, decelerations, heart rate among others) [1,17].

However, considering the above differences in competitive levels, it is important to determine the main contributing factors that influence the training load management [19,20]. From a long-term development perspective, managing physical qualities is an important factor in improving a player’s future sporting career [21,22]. Load discrepancies based on starting status may require compensatory training sessions or competitive breaks optimization periods [23,24]. In professional football, Anderson et al. [13] described that the total activity volume (i.e., training and match load), as well as the total distance covered, were not different between starters, fringe players, and nonstarters, while Los Arcos et al. [14] stated that the match load was solely responsible for a higher weekly training load in starters compared to non-starters. Dalen and Lorås [12] reported a large amount of match-related high-speed running and sprint distances across the weekly training schedule for elite young football players. Therefore, the present research aims to examine the evidence-based training load and determine any similarities with the training of sub-elite youth football players. Thus, the main purpose of this study was to compare the weekly training load across a standard microcycle in starters and non-starters of a sub-elite youth football academy.

## 2. Materials and Methods

### 2.1. Participants and Study Design

Table 1 presents the baseline characteristics of the subsample of 60 male football players from a sub-elite Portuguese football academy. A total of 60 young football players aged between 13 and 20 years were analyzed in this prospective, observational, and cross-sectional study. The daily training load was continuously monitored during a 6-week period of the 2019–2020 competitive season. The training data corresponded to a total of 18 training sessions and 324 observation cases (i.e., starters and non-starters with 164 and 160 observations, respectively).

All participants were informed of the aims and risks of the research. The study only includes players whose legal guardian/next of kin had signed the informed consent to participate. The present research was conducted in accordance with the ethical standards of the Declaration of Helsinki. The experimental approach was approved and followed by the local Ethical Committee from the University of Trás-os-Montes e Alto Douro (3379-5002PA67807).

### 2.2. Eligibility Criteria for Training Data

The eligibility for training data was based on previous studies in sub-elite youth football [10,11] considering the following inclusion criteria: (a) young football players aged between 13 and 20 years old [1]; (b) at least five years of competitive experience in football [21]; (c) training files containing at least 35 consecutive minutes of playing time on the pitch [25]; (d) training data considered a competitive one-game per week schedule and complete full training sessions three times a week (~90 min) [10,11]. The exclusion criteria were: (a) total or partial absence from training due to data collection errors, injury events, rehabilitation sessions, individual training sessions, early withdrawal, and/or missing training; (b) football players aged under 13 or over 20 years; (c) the goalkeeper participated in the training session but was excluded from the analysis [1]. The exclusion criteria resulted in the elimination of 36 observation cases.

The players’ starting status was divided into starters (i.e., started the game at least 55% of the games) and non-starters (i.e., started in less than 55% of the games) [13,26]. The average playing time was 73.82 ± 12.08 and 24.06 ± 9.67 min for starters and non-starters, respectively. The number of observations was adjusted by age group, specifically under 15 (U15), under 17 (U17), and under 19 (U19) [10,11]. The number of observations in weekly training data for each age was: U15 (*n* = 102), U17 (*n* = 99), and U19 (*n* = 120). The microcycle included three training sessions per week (~90 min) with the following “match day minus format” (MD): MD-3 (Tuesday), MD-2 (Wednesday), and MD-1 (Friday) [7,8]. The number of observations in weekly training data for each age was: MD-3 (*n* = 41), MD-2 (*n* = 38), and MD-1 (*n* = 44). The average training session consisted of 18 players with a training session and all age groups were trained on an outdoor pitch with official dimensions (FIFA standard; 100 × 70 m). The training sessions were performed on synthetic turf pitches, from 10:00 a.m. to 8:00 p.m., and with similar environmental conditions (14–20 °C; relative humidity 52–66%) [10,11].

### 2.3. Weekly Training Schedule

The sampled training sessions were categorized according to a specific focus, following the discussion with the coaching staff. All sampled training sessions started with a standard warm-up with low-intensity running, dynamic stretching for main locomotive lower limb muscles, technical actions, and ball possession. The overview of weekly training was potentially variable across categories, such as different training modes with an emphasis on game-based situations and sport-specific skills for football-specific exercises [27,28]. The typical weekly training schedule was categorized based on a typical training microcycle published on youth football [29,30].

The MD-3 (Tuesday) highlighted the recovery and technical skills with an emphasis on individual and group tactical actions by 1v1 to 6v6 small- and medium-sized games (SSG/MSG) (physiological set: 75–80% HR_max_). The MD-2 (Wednesday) focused on the sectorial and collective tactical actions of the game model as training containing the use of large sided games (LSG) (i.e., 7v7 to 10v10) and simulated games (i.e., 11v11) with a physiological set of 75–80% HR_max_. The MD-1 (Friday) emphasized goal-scoring situations and tactical schemes (i.e., corners, free-kicks, penalty kicks) (physiological set: 85–90% HR_max_).

### 2.4. Procedures

The young sub-elite football players were monitored using a portable GPS throughout the whole training session duration (STATSports Apex^®^, Northern Ireland) [10,11]. The GPS device provides raw position velocity and distance at 18 Hz sampling frequencies, including an accelerometer (100 Hz), magnetometer (10 Hz), and gyroscope (100 Hz). Each player wore the micro-tech inner mini pocket of a custom-made vest supplied by the manufacturer, which was placed on the upper back between the two shoulder blades. All devices were activated 30 min prior to training data collection to allow clear and acceptable reception of the satellite signal. Respecting the optimal signal for the measurement of human movement, the match data considered eight available satellite signals as a minimum for the observations [31]. The validity and reliability of the global navigation satellite systems (GNSS) were guaranteed as the GPS has been well established in the literature [31,32,33]. The current variables and thresholds should consider a small error of around 1–2% reported in the 10 Hz STATSports Apex^®^ units [31].

### 2.5. Training Load Measures

#### 2.5.1. External Training Load

The external training loads were obtained with time–motion data: total distance (TD) covered (m), average speed (AvS), maximum speed (SPR) (m/s), relative high-speed running distance (rHSR) (m), high metabolic load distance (HMLD) (m), sprinting distance (SPR) (m), dynamic stress load (DSL) (a.u.), number of accelerations (ACC), and number of decelerations (DEC). The number and duration of sprints were also measured (SPR_D and SPR_N, respectively (m)). The GPS software provided information only on the locomotor categories above 5.50 m/s: rHSR (5.5–6.97 km·h^−1^) and SPR (>6.97 km·h^−1^). The sprints were measured by the number and average sprint distance (m). The HMLD is a metabolic variable defined as the distance in meters covered by a player when the metabolic power exceeds 25.5 W·kg^−1^. HMLD variables include all high-speed running, accelerations, and decelerations above 3 m/s m·s^−2^ [31,32,33]. Both acceleration variables (ACC/DEC) considered the number of accelerations and decelerations performed at maximum intensity (>3 and <3 m/s, respectively). The DSL variable was evaluated by a 100 Hz triaxial accelerometer integrated into the GPS device. The sum of the accelerations is presented in the three orthogonal axes of movement (X, Y, and Z planes) in arbitrary units (a.u.) [34]. The high-intensity activity thresholds were adapted from previous studies [1,2].

#### 2.5.2. Internal Training Load

##### Heart Rate–Based Measures

Heart rate was recorded by a 1 Hz short-range telemetry system GARMIM TM HR band (International Inc., Olathe, KS, USA). Maximum heart rate (HR_max_), average heart rate (AvHR), and percentage of HR_max_ (%HR_max_) values were considered for analysis [35,36]. Training impulse was obtained by Akubat TRIMP [37], reporting a team TRIMP whose equation is based on individual data from the players’ TRIMP; however, it was used to calculate the internal load for each player as: Akubat TRIMP = Training duration × 0.2053e^3.5179x^, among which the HR_ratio_ is the same in Banisters TRIMP [1], e = Napierian logarithms, 3.5179 is the e exponent, and x = HR_ratio_ [37]. HR_max_ was obtained by the Yo Yo intermittent recovery test level 1 (YYIR1) [38].

##### Perceived Exertion and Recovery

The perceived exertion was measured using the 15-point Portuguese Borg Rating of Perceived Exertion 6–20 Scale (Borg RPE 6–20) [39]. The sRPE was obtained by multiplying the total duration of training sessions for each individual RPE score (sRPE = RPE × session duration) following a scale from 6 to 20 [40]. To monitor recovery, each player was asked to report the total quality recovery (TQR) score on a scale from 6 to 20. This scale was proposed by Kenttä and Hassmén [41] to measure the athletes’ recovery perceptions. RPE and TQR were individually collected approximately 30 min before and after each training session, respectively. Players were already familiarized with the procedures and the perceived data were collected using Microsoft Excel^®^ spreadsheet (Microsoft Corporation, Redmond, WA, USA). Previous research has included both scales to examine perceived stress and fatigue in youth football [10,11].

### 2.6. Statistical Analysis

Robust estimates of a 95% confidence interval (CI) and data heteroscedasticity were calculated using randomly 1000 bootstrap samples [11,42]. Data are presented as the mean ± standard deviation (SD), mean differences (Δ) are presented in absolute values, and statistical significance was set at *p* < 0.05. Differences in the players’ starting status were tested with an independent sample *t*-test [43]. Effect sizes (ES) were calculated based on Cohen’s *d* and classified as: 0.2, trivial; 0.6, small; 1.2, large; and >2.0, very large [42,43]. A repeated-measure ANOVA was applied to compare the differences and interactive effects between playing time, session duration, and player’s starting status in the weekly training load [44,45]. Data sphericity was checked by Mauchly’s statistic, and where violated, a Greenhouse–Geiser adjustment was applied. For ANOVA, the ES was computed by the eta square (η^2^) and interpreted as: 0 < η^2^ ≤ 0.04, without effect; 0.04 < η^2^ ≤ 0.25, minimum; 0.25 < η^2^ ≤ 0.64, moderate; and η^2^ > 0.64, strong [46,47]. A comparison of data visualization between starters and non-starters was performed by a violin diagram with a boxplot element (ggplot2). All statistical analyses and data visualization were conducted using JASP software (JASP Team, 2019; version 0.16.3, jasp-stats.org) [43].

## 3. Results

### Weekly Training Load According to the Player’s Starting Status

The descriptive statistics of weekly training load according to the player’s starting status are presented in Table 2.

Table 3 presents the mean comparison between starters and non-starters for external and internal training loads. Only the TD covered presented a significant difference with a moderate effect when comparing between the player’s starting status (*t* = −2.38, Δ = −428.03 m, *p* = 0.018, *d* = 0.26). Training volume was higher for non-starters than starter players (TD_Starters_ = 5105.53 ± 1684.22 vs. TD_Non-starters_ = 5105.53 ± 1684.22 m). Neither the measures of external training intensity nor the internal training load showed significant differences. However, the high intensity showed a trend towards higher values in non-starters. 

**Figure 1:**
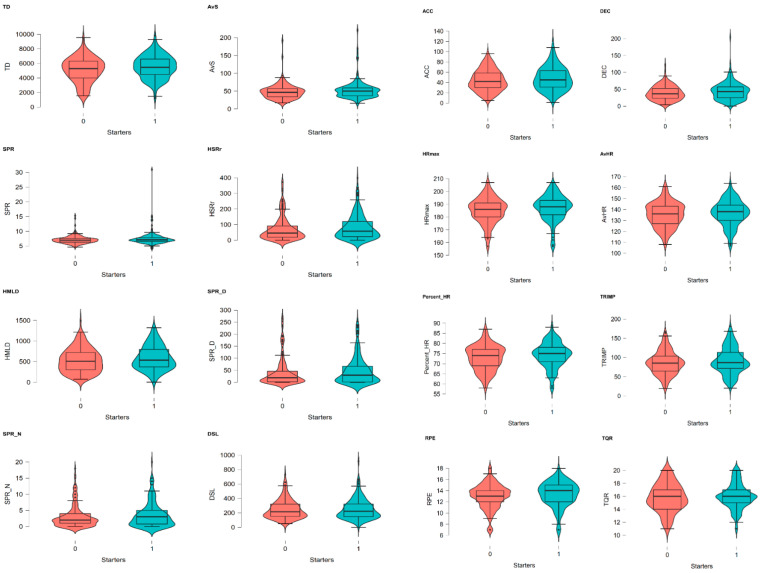
Comparison between starters and non-starters for each training load measure. Note: “Starters” coded 1 (red graph) and “non-starters” coded 2 (green graph).

When considering the playing time and session duration as co-variables, to compare the weekly training load in starters and non-starters, there were significant interactive effects between players’ starting status, playing time, and session duration in overall training load variables, either for within-subjects (F = 140.46; η^2^ = 0.85; *p* < 0.001) or for between-subjects (F = 11.63 to 160.70; η^2^ = 0.05 to 0.76; *p* < 0.001). Figure 1 shows the comparison between starters and non-starters for each training load measure.

## 4. Discussion

The main objective of this study was to compare the weekly training load across a standard microcycle in starters and non-starters of a sub-elite youth football academy. In general, the presented data suggested a trend towards a higher weekly training load in non-starting football players. Additionally, the external and internal training intensity did not seem to differ between the starting status of sub-elite youth football players. However, when considering the co-variance of the playing time and session duration, a significant interactive effect between the players’ starting status, playing time, and session was reported in the overall training load variables.

In this study, only the TD covered seems to be influenced by the player’s starting status in the young sub-elite, with a higher training volume for non-starters compared to starters (moderate effect). A possible explanation may be that coaches tend to prioritize complementary training to equalize training volume and emphasize similar practice opportunities for non-starters [23,24]. The fact that this sub-elite academy of training football only trains three times a week may represent that one of them might represent recovery training for the starters and compensatory training for the non-starters. The current findings are contrary to the evidence produced on the influence of the player’s starting status for elite youth training. In youth elite football, Dalen and Lorås [12] determined a higher average weekly physical load for starters than non-starters in total covered distance, Banister’s TRIMP, accelerations, and sprints. Furthermore, starters completed more moderate and high-intensity running than non-starters and fringe players in professional football [13]. Both training load analyses were performed during the in-season phase as in the present study [12,13]. On the contrary, this study determined that the non-starters covered more distance across the standard microcycle than starters. Current research also suggests a trend towards high-intensity activity as current training data showed a tendency towards higher values in non-starters, specifically for DEC, HSR, and SPR. The weekly training load disparities between elite and sub-elite football players are due to expertise level, periodization strategy, and training content [48,49], considering that it is possible that shorter training duration in sub-elite contexts may lead coaches to prioritize equity of practice opportunities for non-starters [48]. Otherwise, the intra- and inter-individual variation training load may influence the perceived exertion, pacing strategies, and high-intensity demands [11]. In addition, previous studies have demonstrated that non-starter players tend to have higher training workloads, which may result in overreaching, overtraining syndrome, and poor performance [44,45]. This evidence may also be due, in part, to the influence of maturational and motor development factors on the weekly training load [10,11]. Most importantly, the weekly training load across a standard microcycle should consider the co-variance of the playing time and session duration. This is because a non-starter may have 45 min, as well as a starter, since the players’ starting statuses were based on the percentage of started matches and not on the playing time [13,26]. However, this evidence moves in the same direction as the weekly in-season training load verified in professional football players by Los Arcos et al. [14]. According to the study by Los Arcos, although a greater tendency towards a higher perceived exertion-based load for the starters was observed, only the match load was identified as a major factor contributing to a higher weekly training load. In the present study, the perceived exertion tended to be higher for starters than non-starters, for RPE, sRPE, and TQR. Previous studies have demonstrated that the perceived exertion does not seem to show differences either in age group or in maturity status [11]. Given this, the same assumptions seem to occur when considering the player’s starting status as an influential factor in the accumulated training load [1]. All HR-based measures showed no statistical differences between starters and non-starters. However, similar to external training intensity, internal training intensity tends to be higher in non-starters. More specifically, non-starters have higher values for HR_max_ and %HR_max_. Teixeira et al. [11] described higher HR_max_ and Akubat TRIMP in U17, as well as %HR_max_, RPE, and sRPE in U15 sub-elite football players. The current weekly training load showed no differences for Akubat TRIMP between starters and non-starters. Although HR-based measures continue to be useful for training load monitoring, the limitations of measuring high-intensity movements are highly dependent on anaerobic components that have been widely described in the literature [1,2]. The standardization of the application of TRIMP methods to youth sub-elite football players should be considered to alleviate these problems [37]. Additionally, there is a need to reduce the dimensionality of the biomechanical and physiological datasets for a better understanding of the training load [11].

The current study presents some limitations that should be taken into consideration when interpreting and extending the results. First, the training load analysis included only one sub-elite football academy, so the applicability of the results must consider this specificity. Second, quantifying a weekly training load across a standard microcycle should also consider other influencing factors such as periodization structure and match-related contextual factors [10,11,50]. However, the current analysis did not include match data and, consequently, training and match load relationships [1]. The difference between recovery and compensatory sessions from other training days was also not analyzed [10]. Moreover, the training load was extracted from a complete training session, so that in the future the different training exercises should be subdivided to assess the task constraints and modality (i.e., fractional or continuous) such as SSG, high-intensity interval training (HIIT), and simulated game situations [1,51]. Pacing strategies and collective behavior should be considered in future research when analyzing the role of the starting status in match load [20,26,49]. In addition, future research should consider the relationship between compensatory training sessions with match load in youth sub-elite football, as this is an emerging research topic that has not yet been explored in sub-elite training contexts. Additionally, it is still necessary to compare how the behavior of sub-elite and elite football players differs in specific training drills and constrained tasks [1,10,11]. The lack of access to raw positional data made it challenging to perform the fragmented analysis of the entire training session [49]; therefore, future research should focus on physical, physiological, and technical–tactical analysis with an emphasis on comparing starters and non-starters [49,51]. Hence, more analyses are needed for this purpose with a broader follow-up, given the small sample and size of this prospective, cross-sectional, and observational study design. Research on the weekly training load with an integrative performance perspective should also be considered, as key technical and tactical indicators were not explored in this analysis [49].

## 5. Conclusions

The current research suggests a trend toward a higher weekly training load in non-starters, contrary to the published literature to date. The player’s starting status only seems to influence the training volume in sub-elite youth football, unless the covariance of the playing time and session duration are considered. Thus, coaches seem to prioritize complementary training to equalize training volume and emphasize similar practice opportunities for non-starters. Future studies should evaluate the gap between training and match load in this comparison between starters and non-starters.

## Figures and Tables

**Table 1:** Description of the participants’ subsamples according to the player’s starting status.

Variables	Starters (*n* = 164)	Non-Starters (*n* = 160)	Total (*n* = 324)
Age (y)	15.06 ± 1.85	15.33 ± 1.65	15.20 ± 1.75
Height (m)	1.74 ± 0.80	1.73 ± 0.69	1.73 ± 0.08
Weight (kg)	62.64 ± 10.46	62.31 ± 9.57	62.48 ± 10.03
BMI (Kg/m^2^)	20.64 ± 2.01	20.58 ± 2.27	20.61 ± 2.14
Playing time (min)	73.82 ± 12.08	24.06 ± 9.67	49.25 ± 27.21
Session duration/wk (min)	148.13 ± 33.07	175.74 ± 43.52	161.77 ± 38.86

**Table 2:** Mean weekly training load according to the player’s starting status.

Measures	Starters (*n* = 164)	Non-Starters (*n* = 160)
TD (m)	5105.53 ± 1684.22	5533.56 ± 1549.26
AvS (m/min)	48.07 ± 21.02	25.84 ± 16.00
SPR (m/s)	7.12 ± 1.38	7.52 ± 2.45
rHSR (m)	72.52 ± 77.88	81.53 ± 77.96
HMLD (m)	528.48 ± 289.14	588.58 ± 289.02
SPR_D (m)	41.26 ± 59.27	48.26 ± 57.39
SPR_N (n)	2.99 ± 3.51	3.49 ± 3.84
DSL (a.u.)	249.22 ± 130.66	252.30 ± 139.84
ACC (n)	44.14 ± 20.21	47.81 ± 22.83
DEC (n)	39.09 ± 20.97	43.85 ± 26.29
HR_max_ (bpm)	185.03 ± 10.00	186.89 ± 10.12
AvHR (bpm)	135.15 ± 11.04	136.78 ± 11.43
%HR_max_ (bpm)	72.87 ± 6.04	74.20 ± 6.13
Akubat TRIMP (a.u.)	86.05 ± 29.71	91.26 ± 34.07
RPE (a.u.)	12.99 ± 2.18	13.36 ± 2.18
sRPE (a.u.)	1169.45 ± 196.25	1202.06 ± 196.08
TQR (a.u.)	15.80 ± 2.17	15.99 ± 1.91

Abbreviations: ACC—acceleration; AvS—average speed; DEC—deceleration; HMLD—high metabolic load distance; RPE—ratings of perceived exertion; SPR—sprint distance; SPR_N—number of sprints; SPR_D—distance covered at sprinting; sRPE—session ratings of perceived exertion; TD—total distance; TQR—total quality recovery.

**Table 3:** Mean differences between starters and non-starters in the weekly training load.

Variables	*t*-Test				Cohen’s *d*
Measures	*t*	Δ	*p*	*d*	Qualitative Effect
TD (m)	−2.38	−428.03	0.018	0.26	Moderate
AvS (m/min)	−1.88	−4.90	0.062	0.21	Moderate
SPR (m/s)	−1.81	−0.40	0.071	0.20	Moderate
rHSR (m)	−1.09	−9.43	0.277	0.12	Small
HMLD (m)	−1.87	−60.10	0.062	0.21	Moderate
SPR_D (m)	−1.08	−7.00	0.281	0.12	Small
SPR_N (n)	−1.23	−0.50	0.222	0.14	Small
DSL (a.u.)	−0.21	−3.08	0.838	0.02	Small
ACC (n)	−1.53	−3.67	0.126	0.17	Small
DEC (n)	−1.80	−4.76	0.072	0.20	Moderate
HR_max_ (bpm)	−1.67	−1.86	0.096	0.19	Small
AvHR (bpm)	−1.30	−1.63	0.193	0.15	Small
%HR_max_ (bpm)	−1.97	−1.33	0.049	0.22	Moderate
Akubat TRIMP (a.u.)	−1.47	−5.21	0.143	0.16	Small
RPE (a.u.)	−1.50	−0.36	0.136	0.17	Small
sRPE (a.u.)	−1.50	−32.61	0.136	0.17	Small
TQR (a.u.)	−0.86	−0.20	0.392	0.10	Small

Abbreviations: Δ—mean differences; ACC—accelerations; ALL—overall independent position group; AvS—average speed; bpm—beat per minute; CD—central defenders; CM—central midfielders; DEC—decelerations; FB—fullbacks; FW—forwards; rHSR—relative high speed running; SPR—sprints; TD—total distance; WM—wide midfielders.

## Data Availability

Data are available under request to the contact author.
